# Evaluating immunoreactivity of hybridoma-derived antibody mixtures generated against AU-565 cell line for diagnosis and immunotherapy of breast cancer

**DOI:** 10.1186/s12885-026-16143-7

**Published:** 2026-05-09

**Authors:** Murat Ihlamur, Atıfcan Demi̇rci̇oğlu, Aslı Pınar Zorba, Emrah Şefik Abamor, Melahat Bağırova, Adil Allahverdi̇yev

**Affiliations:** 1 Department of Bioengineering, Faculty of Chemical and Metallurgical Engineering, Yildiz Technical University, Istanbul, Turkey; 2 Department of Electronics and Automation, Vocational School, Biruni University, Istanbul, Turkey; 3 Department of Medical Services and Techniques, School of Vocational of Healty, Istinye University, Istanbul, Turkey; 4 Scientific Research Institute of Medical Prevention named after V. Akhundov, Baku, Republic of Azerbaijan; 5 Department of Biotechnology, Baku Engineering University, Baku, Republic of Azerbaijan

**Keywords:** Breast cancer, Hybridoma-derived antibody mixture, Cytokine analysis, Complete Freund’s Adjuvant, Diagnostic immunoassay

## Abstract

**Background:**

This study aimed to generate antibodies against the whole-cell lysate of the AU-565 breast cancer cell line using hybridoma technology and to assess their immunogenic properties and preliminary immunoassay-oriented reactivity. Two adjuvants, Complete Freund’s Adjuvant (CFA) and Polyoxidonium (PO), were compared for their ability to enhance immune responses. Cytokine profiling was also performed to evaluate the immunomodulatory effects of antigen–adjuvant formulations.

**Methods:**

AU-565 cell lysates were prepared by sonication and combined with CFA or PO. Immunostimulatory activity was evaluated in vitro by nitric oxide (NO) production in J774 macrophages and in vivo by antibody titers in Balb/c mice. Hybridoma cells were generated from splenocytes of immunized mice. Since single-cell cloning was not performed, antibodies were collected from pooled hybridoma cultures, representing a mixture of monoclonal antibodies recognizing multiple epitopes. Cytokine levels (IL-6, IL-12, GM-CSF, and TNF-α) were quantified by ELISA to characterize the inflammatory response pattern induced by the antigen–adjuvant formulations.

**Results:**

The AU-565 antigen + CFA formulation significantly increased NO production compared with untreated control macrophages and generated the strongest antibody titers compared with the PBS control group. Cytokine profiling showed that the AU-565 antigen + CFA formulation significantly increased IL-12, GM-CSF, and TNF-α secretion compared with untreated control macrophages, indicating a pronounced pro-inflammatory response pattern in the macrophage model, while a moderate IL-6 increase relative to untreated control macrophages suggested partial support for humoral-associated signaling. The hybridoma-derived antibody mixture displayed stronger ELISA reactivity toward AU-565 and MCF-7 lysates than the commercial antibody, indicating broader relative lysate recognition under the assay conditions used.

**Conclusion:**

The hybridoma-derived antibody mixture showed strong immunogenicity, cytokine-associated immune stimulation, and preliminary potential for breast cancer-oriented immunoassay development; however, additional validation of specificity and analytical performance is required before diagnostic utility can be concluded. The AU-565 antigen–CFA combination not only enhanced antibody production but also promoted a favorable cytokine profile, underscoring CFA’s superiority as an adjuvant in breast cancer-oriented immunogen design.

**Graphical Abstract:**

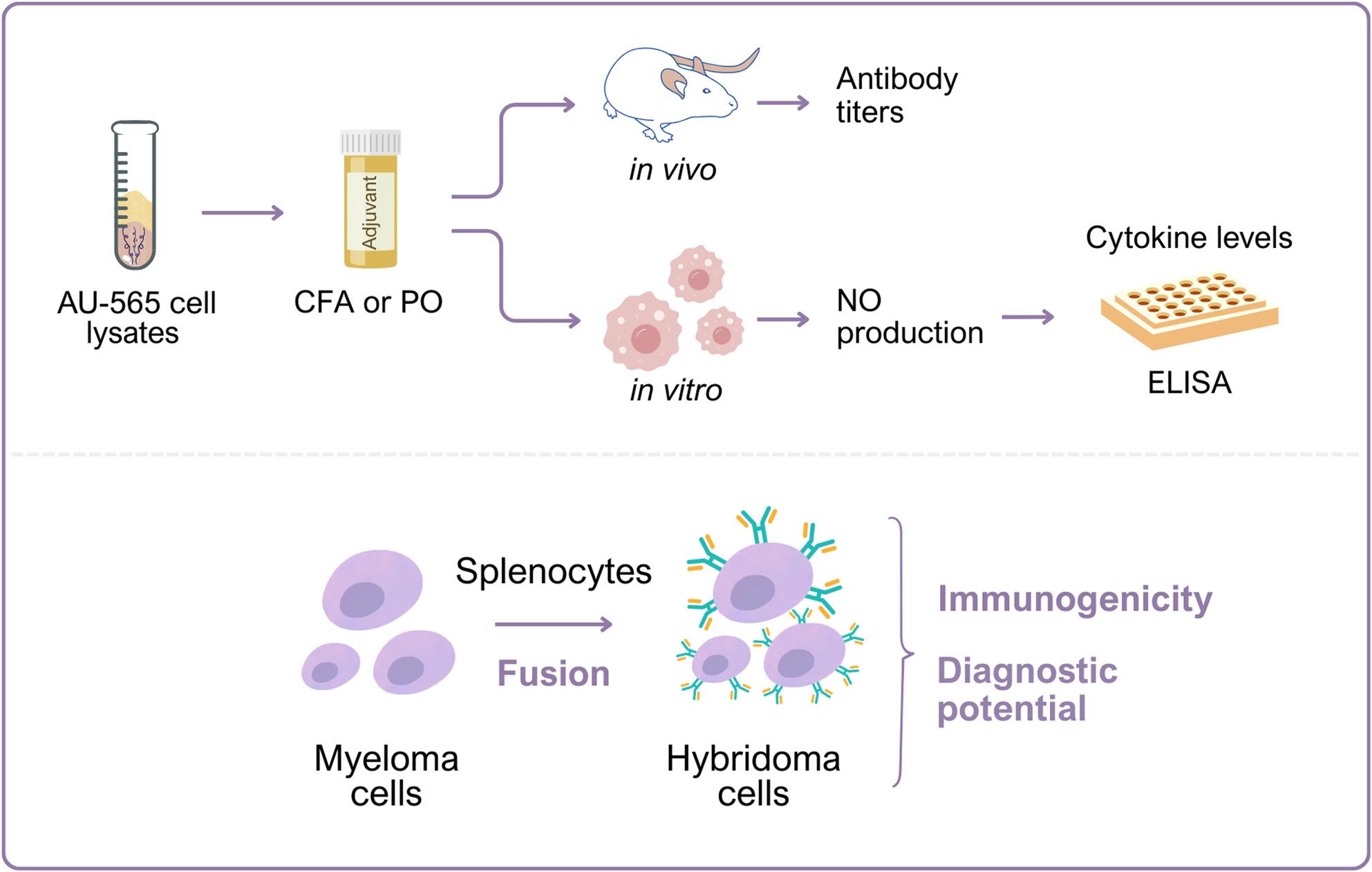

## Introduction

Cancer is characterized by the uncontrolled proliferation of cells, and among its various types, breast cancer remains the most frequently diagnosed malignancy in women worldwide, with over 2 million new cases reported in 2020. It also ranks as the second leading cause of cancer-related deaths among women. The global burden of breast cancer is expected to rise significantly, reaching an estimated 2.7 million new cases and 870,000 deaths by 2030 [1]. While conventional treatment strategies such as surgery, chemotherapy, and radiotherapy are commonly employed, recent advancements in targeted therapies and immunotherapy have opened promising avenues for improved clinical outcomes [2]. Immunotherapy, in particular, aims not only to eliminate cancer cells but also to induce long-term immune memory that prevents disease recurrence. By presenting tumor antigens to B and T lymphocytes, this approach stimulates both cellular and humoral responses, providing therapeutic and prophylactic benefits. However, despite notable progress, no effective and standardized vaccine formulation has been approved for breast cancer to date [3]. Another pressing challenge is the limitation of current diagnostic tools. Mammography and ultrasound are routinely used but suffer from low sensitivity, false positive and false negative results, and limited detection of early-stage or certain subtypes of breast tumors [4, 5]. Consequently, there is an urgent need for the development of new, sensitive, and specific diagnostic platforms. Breast cancer is highly heterogeneous at both inter-patient and intra-tumoral levels, which directly impacts antigen expression patterns and limits the performance of narrowly targeted diagnostic reagents [6]. Molecular and phenotypic heterogeneity has been widely documented across breast cancer cell lines and subtypes, indicating that broad epitope coverage may be required for improved biomarker capture and immunoassay sensitivity [7]. Therefore, antibody preparations capable of recognizing multiple epitopes can be advantageous when the target antigen landscape is diverse or partially unknown, particularly in whole-cell lysate-based strategies where multiple tumor-associated antigens coexist [8].

**Fig. 1:**
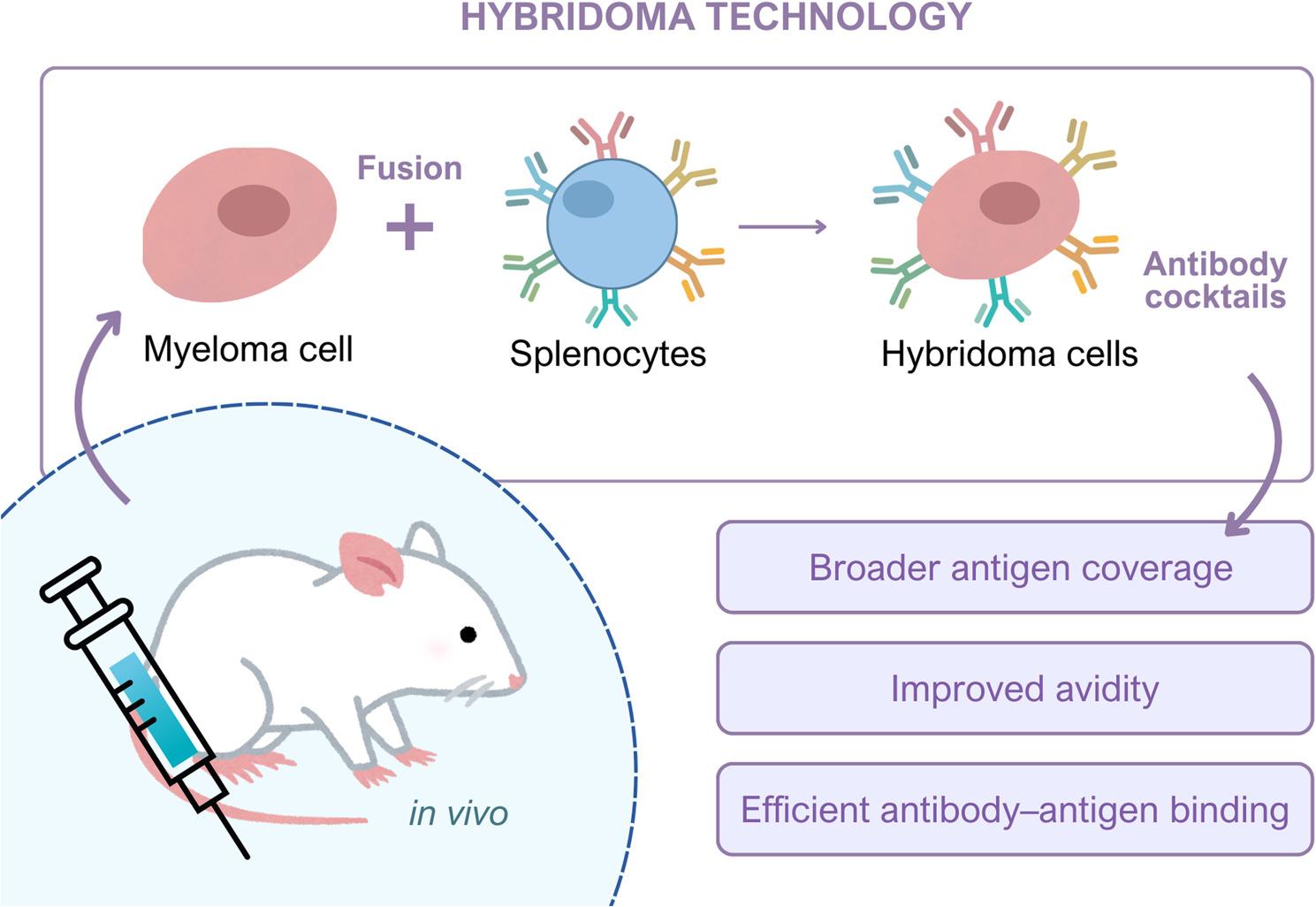
Schematic overview of hybridoma technology and generation of pooled hybridoma-derived antibody mixtures. Splenocytes from immunized mice are fused with myeloma cells to generate hybridoma cells. When single-cell cloning is not performed and hybridoma cultures are maintained as pooled populations, the resulting supernatants contain a mixture of monoclonal antibodies recognizing multiple epitopes, enabling broader antigen coverage and potentially improved functional avidity

Antibodies generated through hybridoma technology are typically associated with monoclonal antibody (mAb) production, offering high specificity through epitope selectivity [9]. However, when hybridoma cultures are maintained without single-cell cloning, multiple antibody-secreting hybridoma populations may coexist, resulting in a pooled antibody output that can recognize multiple epitopes [10]. Such hybridoma-derived antibody mixtures may provide broader antigen coverage and improved avidity when targeting heterogeneous tumor-associated antigens [11]. However, in the absence of single-cell cloning, pooled hybridoma cultures may also introduce compositional variability, clonal drift during long-term culture, and reduced batch-to-batch reproducibility compared with isolated monoclonal clones. This workflow and the rationale for obtaining multi-epitope antibody mixtures from pooled hybridoma populations are summarized in Fig. 1.

Importantly, the concept of using multi-antibody preparations has gained increasing attention in oncology, where oligoclonal antibody mixtures or antibody cocktails may provide improved functional avidity and broader antigen recognition compared with single-epitope binding antibodies [12]. This rationale is also consistent with current immune-target discovery frameworks in cancer immunotherapy, which emphasize that tumor heterogeneity, immune escape mechanisms, and signaling diversity often require broader target recognition strategies rather than reliance on a single molecular epitope [13]. In this context, pooled hybridoma-derived antibody mixtures capable of recognizing multiple lysate-associated epitopes may offer a conceptually relevant experimental model for capturing complex tumor-associated antigen landscapes, although their analytical and functional validation remains necessary. International terminology also recognizes “antibody cocktails/mixtures” as pooled antibody products composed of multiple monoclonal specificities. In this context, pooled hybridoma supernatants may be viewed as a practical experimental analogue of an oligoclonal antibody mixture, potentially improving antigen capture in heterogeneous tumor models [14].

In hybridoma-based antibody production, the choice of adjuvant plays a critical role in modulating antigen immunogenicity and, consequently, the quality and quantity of the resulting antibodies. Complete Freund’s Adjuvant (CFA), a widely used bacterial-based immunostimulant, and Polyoxidonium (PO), a polymer-based adjuvant with lower toxicity, are among the adjuvants of interest in vaccine design [15–18]. Although CFA is traditionally considered more potent, its potential toxicity remains a concern, whereas PO offers a safer profile with growing application in immunization studies.

Despite extensive literature on monoclonal antibodies and adjuvant selection, studies investigating hybridoma-based antibody generation against whole breast cancer cell lysates—particularly under non-clonal conditions resulting in pooled hybridoma antibody mixtures—and evaluating the influence of CFA or PO adjuvants remain limited. Notably, the present study combines (i) whole-cell lysate immunogen design, (ii) comparative adjuvant evaluation (CFA vs. PO), and (iii) pooled hybridoma antibody mixture generation to address antigen heterogeneity in breast cancer. This integrated workflow provides a practical experimental platform for developing antigen-capture immunoassays with broader epitope coverage. Therefore, the primary objective of this study was to generate a hybridoma-derived antibody mixture (pooled hybridoma supernatant) against the whole-cell lysate of the AU-565 breast cancer cell line and to evaluate the comparative immunogenic effects of two distinct adjuvants, CFA and PO, and the preliminary immunoassay-oriented reactivity of the resulting pooled hybridoma-derived antibody preparation. We present a practical workflow that combines whole-cell lysate immunization with adjuvant benchmarking and pooled hybridoma supernatant–based multi-epitope antibody generation, and we demonstrate its cross-line reactivity (AU-565 and MCF-7) to support broad antigen capture in heterogeneous breast cancer models.

## Materials and methods

### Cell culture

AU-565 human breast cancer cells (HER2-positive) were obtained from the American Type Culture Collection (ATCC, Manassas, VA, USA). AU-565 cells were cultured in RPMI-1640 medium containing 10% fetal bovine serum (FBS) and 1% penicillin–streptomycin. L929 fibroblast cells and J774 murine macrophage cells, also obtained from ATCC, were maintained under the same culture conditions. All cultures were incubated at 37 °C in a humidified atmosphere containing 5% CO₂. The P3-X63-Ag8.653 murine myeloma cell line used for hybridoma fusion was grown in RPMI-1640 medium supplemented with 20% FBS and 20 µg/mL 8-azaguanine (Sigma-Aldrich, USA) [19]. Cells between passages 10 and 15 were used throughout the experiments.

### Preparation of breast cancer antigens

AU-565 cells were collected when they reached approximately 90% confluency and washed with cold PBS. Cell lysates were then prepared on ice using a probe sonicator (Sonics VCX130) for five cycles of 40 s at 40% amplitude. After sonication, the lysate was centrifuged at 10,000 rpm for 3 min, and the supernatant fraction was used as the antigen preparation. Protein concentration was measured by the Warburg–Christian method with absorbance readings at 260/280 nm using a UV–Vis spectrophotometer [20]. For immunization experiments, 100 µg of lysate-derived antigen was administered to each mouse in a final injection volume of 0.2 mL. Total protein yield from each lysate batch and endotoxin contamination were not separately determined in this study.

### Nitric oxide assay

Nitric oxide (NO) release was determined by the Griess assay in order to assess the immunostimulatory activity of the prepared formulations in J774 macrophages. Cells were seeded at a density of 1 × 10⁵ cells/mL and cultured for 24 h at 37 °C under 5% CO₂. After this incubation, macrophages were exposed to sonication-derived antigen at concentrations of 10, 20, 40, 80, 100, 120, and 160 µg/mL, either alone or together with adjuvants. Complete Freund’s adjuvant and PO were used at a concentration of 40 µg/mL. Untreated J774 macrophages cultured in complete medium were included as the negative control for basal NO production. Following 48 h of treatment, culture supernatants were collected and reacted with Griess reagent. After 10 min of incubation at room temperature, absorbance was recorded at 540 nm using an ELISA plate reader [21].

### Cell viability assay

The cytotoxic effect of antigen and antigen–adjuvant formulations was evaluated using the MTT assay. L929 fibroblast cells were incubated for 48 h with sonication-derived antigen at 10, 20, 40, 80, 100, 120, and 160 µg/mL. The same antigen concentrations were also tested in combination with Complete Freund’s adjuvant or PO, each used at 40 µg/mL. Untreated L929 cells maintained in complete culture medium served as the negative control and were defined as representing 100% viability. After treatment, MTT solution was added to the wells, and the plates were kept for 3 h at 37 °C in the dark. The culture medium was then removed, and the formed formazan crystals were dissolved in DMSO. Absorbance was measured at 570 nm with a microplate reader, and relative cell viability was calculated accordingly [22].

### In vitro cytokine quantification

To evaluate the cytokine responses (IL-6, IL-12, GM-CSF and TNF-α) induced by the developed vaccine formulations in macrophage cells, commercial ELISA kits were used. Untreated J774 macrophages maintained in complete culture medium were used as the negative control to determine basal cytokine secretion levels. ELISA plates pre-coated with anti-IL-6 antibodies were prepared using six standard concentrations (30 pg/mL, 60 pg/mL, 120 pg/mL, 240 pg/mL, 480 pg/mL, and 960 pg/mL). Similarly, anti-IL-12 plates were prepared with 4 pg/mL to 128 pg/mL standards; GM-CSF plates with 10 pg/mL to 320 pg/mL; and anti-TNF-α plates with 40 pg/mL to 1280 pg/mL. From each standard dilution, 50 µl was transferred into the respective wells. A volume of 40 µl from each vaccine formulation was added to the test wells. Subsequently, 10 µl of biotinylated anti-mouse antibody specific to each cytokine kit was added to the wells, followed by the addition of 50 µl Streptavidin-HRP substrate to all wells. Plates were incubated at 37 °C for 1 h to allow streptavidin to bind to biotin. After incubation, plates were washed five times with wash buffer, and 50 µl each of substrate A and substrate B were added. The reaction was incubated in the dark for 10 min. To terminate the enzymatic reaction, 50 µl of stop solution was added. Absorbance was measured at 450 nm using an ELISA reader.

In this study, IL-6, IL-12, GM-CSF, and TNF-α were selected as representative cytokine markers to characterize the inflammatory response pattern induced by AU-565 lysate formulations. IL-12 and TNF-α were selected because they are widely associated with pro-inflammatory signaling and macrophage-mediated activation [23], while GM-CSF was included because of its relevance to myeloid cell stimulation and antigen-presenting cell support [24]. IL-6 was included because it may reflect inflammatory conditions that contribute to humoral-associated immune responses and B-cell support [25]. Collectively, these cytokines were evaluated to provide a preliminary profile of the balance between pro-inflammatory activation and humoral-associated immune signaling induced by the antigen–adjuvant combinations.

### Experimental animals

All animal procedures (vaccination and blood collection) were conducted at the Bezm-i Alem University Experimental Animals Research Center following approval from the Istanbul Bezm-i Alem University Animal Experiments Ethics Committee (approval no. 2018.05). The study design was planned as an exploratory in vivo immunization experiment and included three groups of female Balb/c mice (6 weeks old; *n* = 3 per group), in accordance with institutional ethical considerations for animal use. Prior to immunisation, ~ 0.2 mL of blood was collected from the tail vein for baseline (pre-vaccination) titre determination. These pre-immune serum samples were used as baseline negative controls for antibody titer evaluation. Mice were immunised intraperitoneally with 0.2 mL of the assigned formulation per injection as follows: (i) the PO group received 100 µg sonicated breast cancer antigen mixed with 250 µg Polyoxidonium (PO; Polyoxidonium^®^, NPO Petrovax Pharm), which was supplied as a lyophilized preparation and reconstituted immediately before administration; (ii) the Complete Freund’s group received a 1:1 mixture of 100 µg sonicated breast cancer antigen and 100 µg Complete Freund’s adjuvant [26]; and (iii) the negative control group received phosphate-buffered saline (PBS) vehicle alone. Immunisations were administered at one-week intervals until the antibody response reached approximately 10-fold over the control group. Blood samples were collected prior to each immunisation for titre monitoring. In the final booster, adjuvant was omitted and antigen alone was administered in the immunised groups.

### Euthanasia and tissue collection

Mice were euthanized in accordance with the approved animal study protocol and institutional animal care requirements of the Istanbul Bezm-i Alem University Animal Experiments Ethics Committee (approval no. 2018.05). General anesthesia was achieved by intraperitoneal injection of ketamine and xylazine at doses of 100 mg/kg and 10 mg/kg, respectively. Before cervical dislocation, the depth of anesthesia was verified by the absence of pedal withdrawal and toe-pinch responses. Death was confirmed by the lack of respiration, heartbeat, and reflex activity. After confirmation of death, spleens were removed immediately for hybridoma fusion procedures, and peritoneal macrophages were collected for use as feeder cells.

### Cell fusion and cloning

Peritoneal macrophages were prepared as feeder cells using 10-week-old Balb/c mice from the PBS control group. Mice were humanely euthanised under ketamine–xylazine anaesthesia followed by cervical dislocation, and peritoneal macrophages were harvested in RPMI-1640. For hybridoma generation, a Balb/c mouse exhibiting an ELISA titre approximately 10-fold higher than the control was euthanised using the same procedure, and the spleen was collected immediately. Splenocytes were fused with myeloma cells at a 10:1 ratio using PEG1500 (Sigma-Aldrich) and cultured in RPMI-1640 supplemented with 20% FBS. Following fusion, cells were subjected to HAT selection and plated into 96-well plates, then incubated at 37 °C with 5% CO₂. After ~ 14 days, supernatants were screened for antibody production, after which cultures were maintained in HT medium and antibody levels were monitored from hybridoma supernatants at defined intervals [27]. After fusion and selection, hybridoma cultures were maintained as pooled populations without single-cell cloning (e.g., limiting dilution). Therefore, antibodies collected from culture supernatants represented a mixture of monoclonal antibodies secreted by multiple hybridoma clones rather than a single monoclonal antibody preparation.

### Enzyme-Linked Immunosorbent Assay (ELISA)

Serum IgG responses specific to the sonication-derived breast cancer antigen were measured by indirect ELISA. The antigen preparation was diluted to 10 µg/mL in 0.05 M carbonate coating buffer (pH 9.6), and 100 µL was added to each well of 96-well plates. Plates were incubated overnight at 4 °C to allow antigen coating. After washing with PBS containing 0.05% Tween-20, nonspecific binding sites were blocked with 2% skimmed milk for 1 h at 37 °C.

Serum samples were diluted 1:50 in PBS/Tween-20 supplemented with 2% milk and added to the antigen-coated wells for 1 h at 37 °C. Pre-immune sera obtained before the first immunization were included as negative controls for antigen-specific antibody detection. After washing, alkaline phosphatase-conjugated anti-IgG secondary antibody diluted 1:1000 was added at 100 µL/well and incubated for 1 h at 37 °C. The plates were then washed again, and p-nitrophenyl phosphate (pNPP; Sigma-Aldrich) was used as the substrate. Color development was carried out for 30 min at room temperature in the dark, and absorbance was measured at 405 nm using a microplate reader [28].

### Statistical analysis

GraphPad Prism 9 software was used for statistical analyses. The data were analyzed using one-way ANOVA with Tukey’s multiple comparisons test. All experiments were performed in at least three independent replicates unless otherwise stated. Data were presented as mean ± standard deviation (SD). Statistical significance was defined as *p* < 0.05.

## Results

### Determination of NO activity

**Fig. 2:**
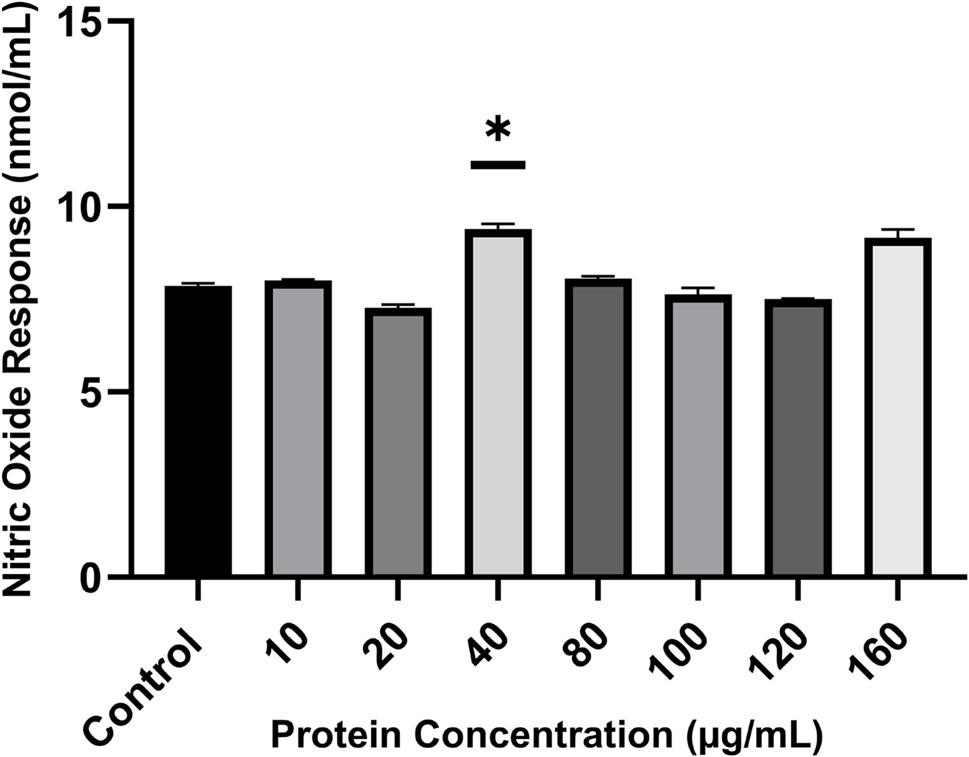
Nitric oxide responses to AU-565 antigen concentration in J774 cell line

Nitric oxide (NO) is an immune-associated mediator involved in antimicrobial defense, redox regulation, and immune signaling [29]. Depending on its concentration, NO may influence the balance between Th1- and Th2-type immune responses; lower levels are generally associated with Th2-skewed responses, whereas higher levels may support Th1-type activation. Based on this background, NO release from J774 macrophages was measured to evaluate the in vitro immunostimulatory potential of the prepared vaccine formulations. Since macrophage-derived NO is commonly used as an indicator of immune activation, this assay was applied as a preliminary screening step for formulation-induced stimulation. Similar increases in NO production have also been reported for other vaccine candidates [30]. Therefore, sonication-derived antigen was first tested at increasing concentrations to identify the antigen dose that produced the most suitable NO response in macrophages. The results indicated that the highest NO level was observed when macrophages were exposed to an antigen concentration of 40 µg/mL. Macrophage-derived nitric oxide (NO) was quantified to evaluate the immunostimulatory capacity of antigen formulations. Treatment with 40 µg/mL AU-565 cell lysate resulted in the highest NO secretion among the antigen-only groups and showed a 1.2-fold increase compared with untreated J774 macrophages (*p* < 0.05, one-way ANOVA with Tukey’s test). The untreated macrophage group served as the negative control and represented basal NO secretion, allowing antigen-induced NO production to be evaluated relative to baseline macrophage activity. This concentration was selected for subsequent adjuvant combination studies (Fig. 2). This response may be explained by an optimal antigenic stimulation threshold at 40 µg/mL. At lower lysate concentrations, the amount of available tumor-associated antigenic components may have been insufficient to maximally activate J774 macrophages. In contrast, higher lysate concentrations may not have further enhanced NO production because excessive lysate-derived cellular components can reduce macrophage responsiveness, alter cell metabolism, or induce a plateau effect in inducible nitric oxide synthase-related activation. Therefore, the peak NO response observed at 40 µg/mL was interpreted as the concentration providing the most favorable balance between antigenic stimulation and cellular tolerance under the present in vitro conditions.

**Fig. 3:**
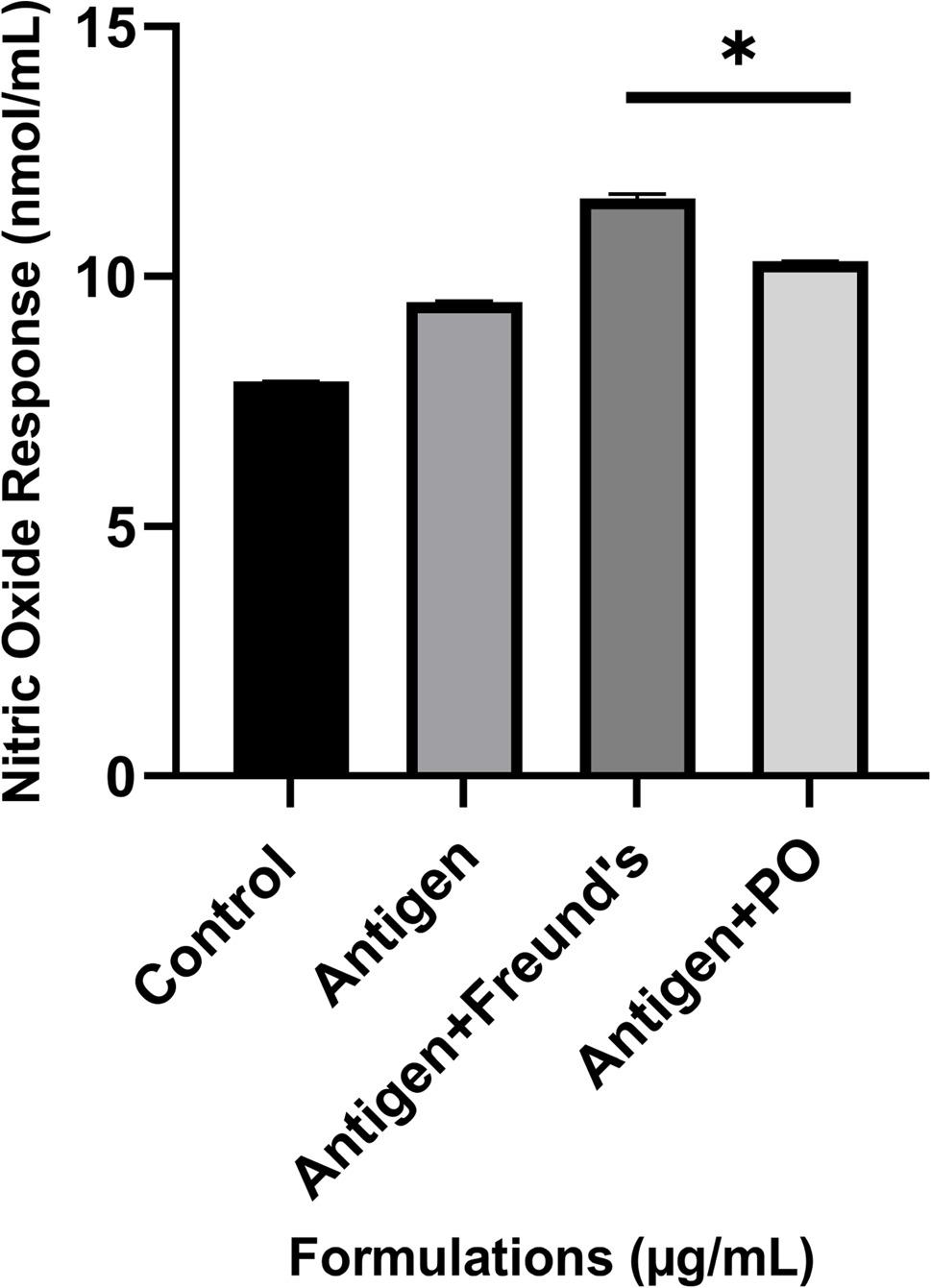
*In vitro* immunostimulatory activity of antigen and adjuvant combinations at a concentration of 40 µg/mL in the macrophage cell culture system

Adjuvants are substances used to enhance the immunogenicity of antigens and promote stronger immune responses following vaccination [31]. Here, we calculated the in vitro immunogenic features of antigen-adjuvant combinations in terms of secreted NO levels from treated macrophages. Antigens were combined with Freund’s and PO adjuvants and stimulatory activities of combinations were compared with sole antigens. It was shown that NO production was significantly higher in macrophages treated with antigen–adjuvant combinations than in macrophages exposed to antigen alone. In this assay, antigen–adjuvant combinations induced higher NO activity than antigen alone. Subsequent testing showed that combining antigen with adjuvants significantly enhanced NO secretion. The AU-565 antigen + CFA formulation yielded a 1.47-fold increase, while the AU-565 antigen + PO formulation showed a 1.3-fold increase, each compared with untreated control macrophages (*p* < 0.05). These results confirm that both adjuvants augmented the immunogenic profile of the lysate, with CFA being superior in NO induction (Fig. 3).

### Cell viability assay

**Fig. 4:**
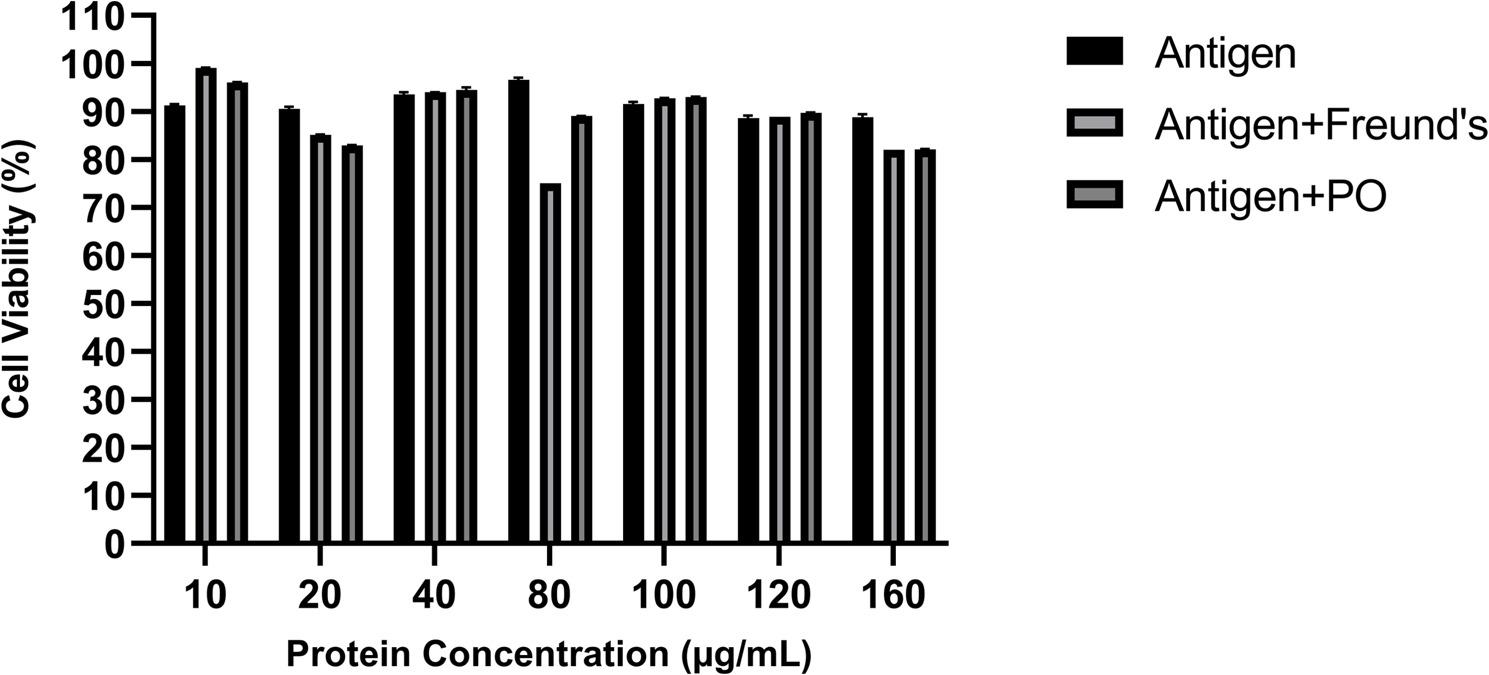
The cytotoxic effect of antigens and antigen-adjuvant combinations on viability in L929 fibroblast cell culture system

The MTT assay was performed to evaluate the biocompatibility of the prepared vaccine formulations. Cell viability was assessed in L929 fibroblast cells after exposure to antigen alone or antigen–adjuvant combinations. Figure 4 shows the viability results of L929 cells treated with the different formulations. The tested antigen concentrations did not markedly reduce L929 fibroblast viability. The formulation containing 40 µg/mL antigen and 40 µg/mL adjuvant was selected for subsequent immunization studies by considering both the NO response observed in J774 macrophages and the viability profile observed in L929 fibroblast cells. Thus, this formulation was considered suitable for further in vivo evaluation. All tested concentrations up to 160 µg/mL showed over 85% cell viability, confirming the non-toxic nature of both antigen and antigen-adjuvant mixtures. Cell viability values were calculated relative to untreated L929 fibroblast cells, which served as the negative control and were accepted as 100% viability. The 40 µg/mL antigen + CFA formulation showed > 95% viability and was selected for animal immunization (Fig. 4). These findings support the safety of the candidate vaccine formulations [32].

### In vitro cytokine production measurement

**Fig. 5:**
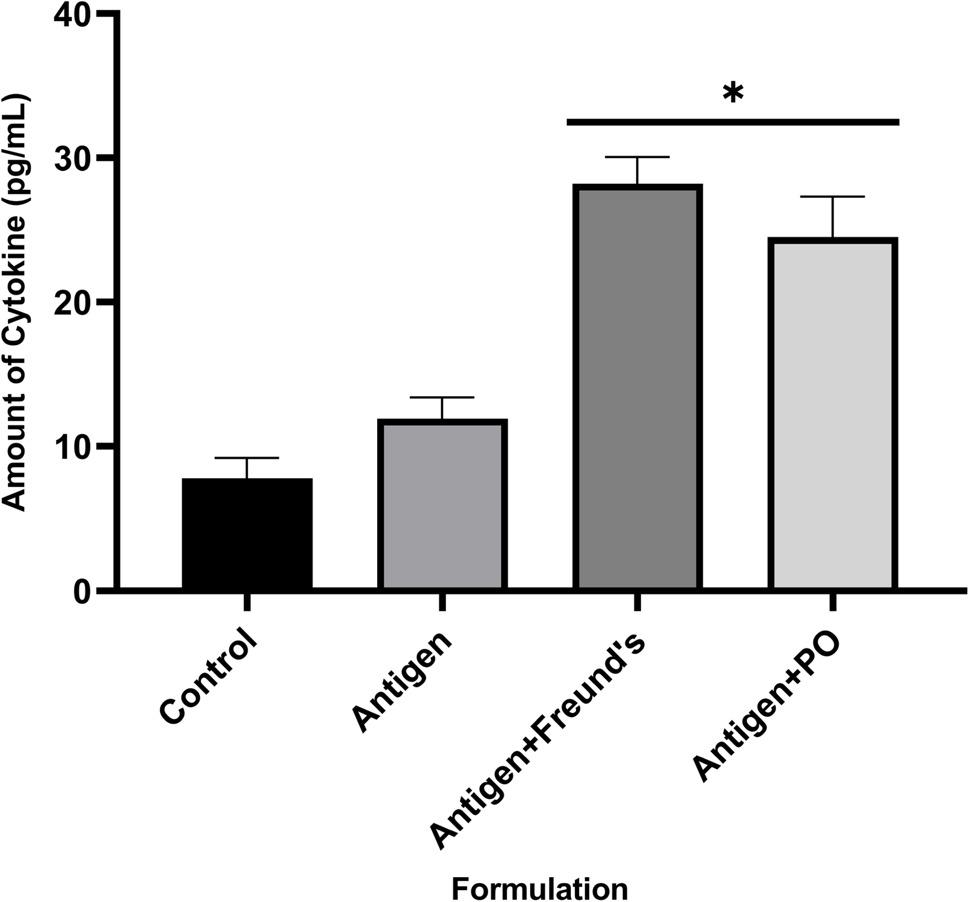
IL-6 cytokine levels in J774 macrophage cells

The levels of cytokines IL-6, IL-12, TNF-α, and GM-CSF produced by J774 murine macrophage cells in response to the prepared formulations were quantified using commercial ELISA kits. Significant increases in cytokine levels were observed in the groups treated with either antigen alone or antigen combined with adjuvants. Untreated J774 macrophages served as the negative control and represented basal cytokine secretion levels, allowing cytokine induction by antigen and antigen–adjuvant formulations to be evaluated relative to baseline macrophage activity. Since the aim of vaccine development is to enhance the immune response by promoting antibody production, high levels of TNF-α, IL-12, GM-CSF and relatively lower increases in IL-6 are considered desirable. The results for IL-6 are presented in Fig. 5. Among the formulations tested, the combination of Antigen with Freund’s induced the highest IL-6 response. Macrophages treated with the Antigen+Freund’s formulation produced 3.61-fold higher IL-6 levels compared to the control group, which was statistically significant (*p* < 0.05).

**Fig. 6:**
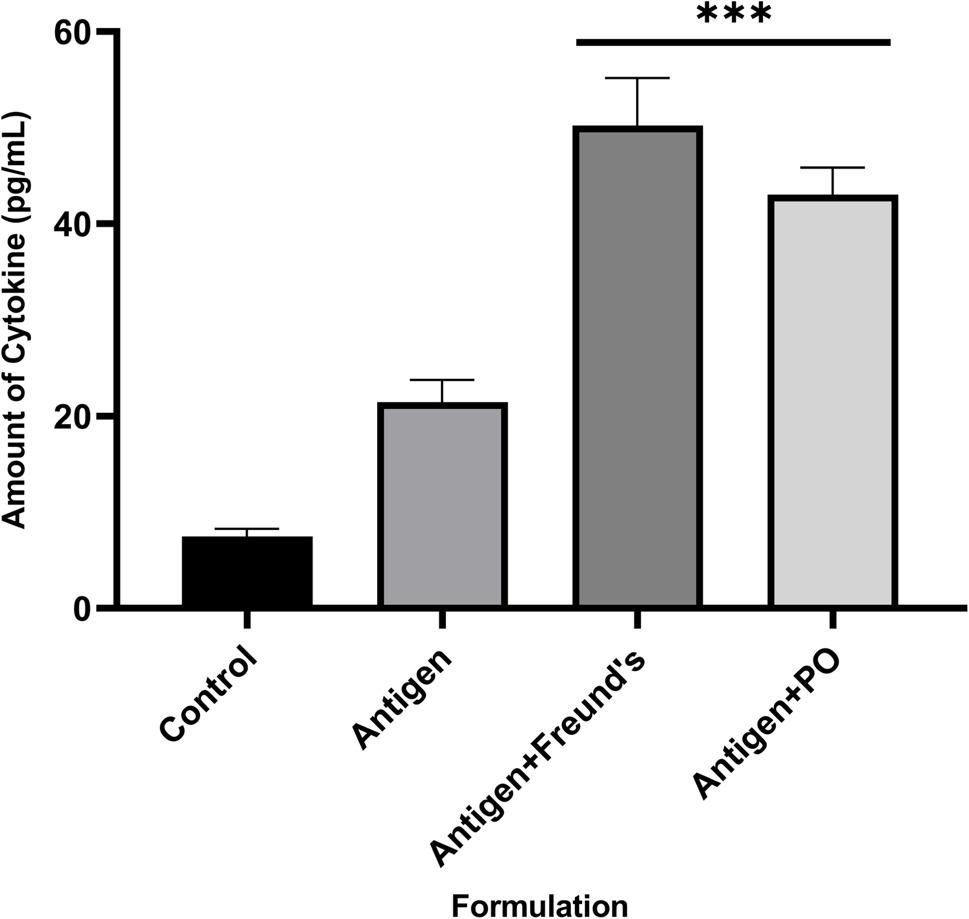
IL-12 cytokine levels in J774 macrophage cells

The levels of IL-12 cytokine produced by J774 macrophage cells in response to the formulations are shown in Fig. 6. Analysis of the data revealed that the combinations of Antigen with Freund’s induced the highest IL-12 levels. Specifically, macrophages treated with either the Antigen+Freund’s formulations produced 6.706-fold higher IL-12 levels compared to the control group, a statistically significant difference (*p* < 0.001).

**Fig. 7:**
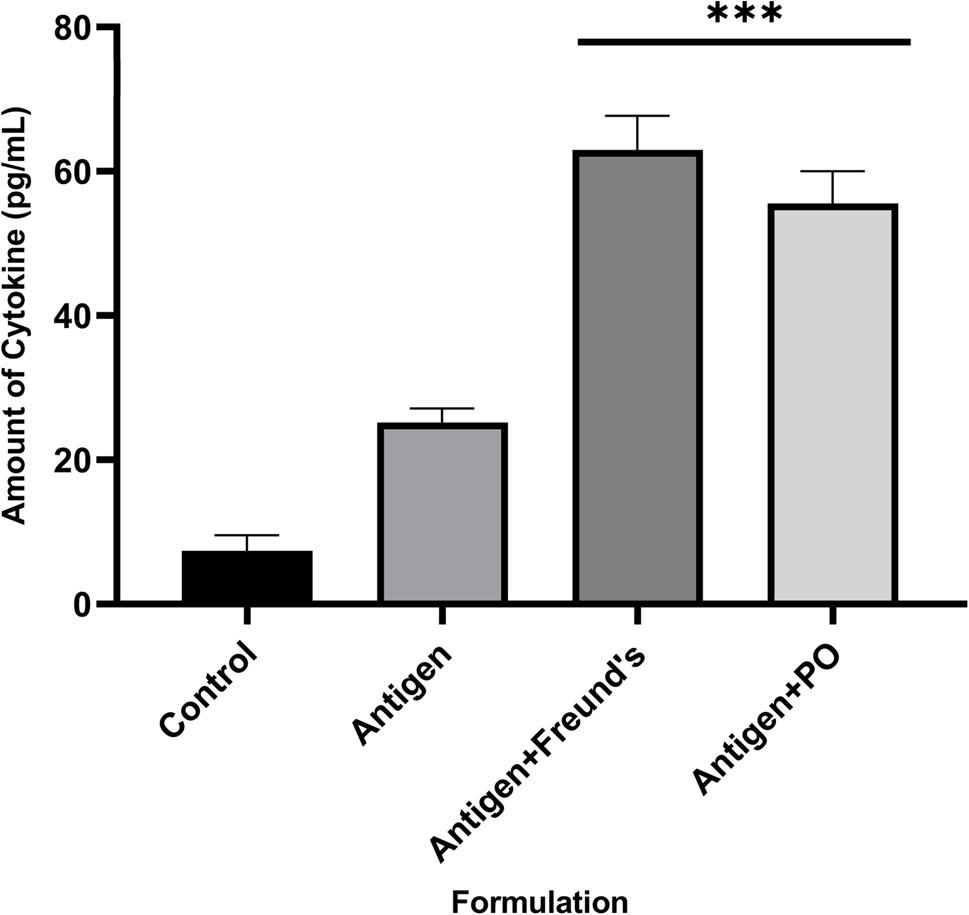
TNF-α cytokine levels in J774 macrophage cells

The TNF-α cytokine levels produced by J774 macrophage cells in response to the formulations are presented in Fig. 7. Analysis of the data revealed that the combination of Antigen with Freund’s induced the highest TNF-α production. Specifically, macrophages treated with the Antigen+Freund’s formulation produced 8.5-fold higher TNF-α levels compared to the control group (*p* < 0.001).

**Fig. 8:**
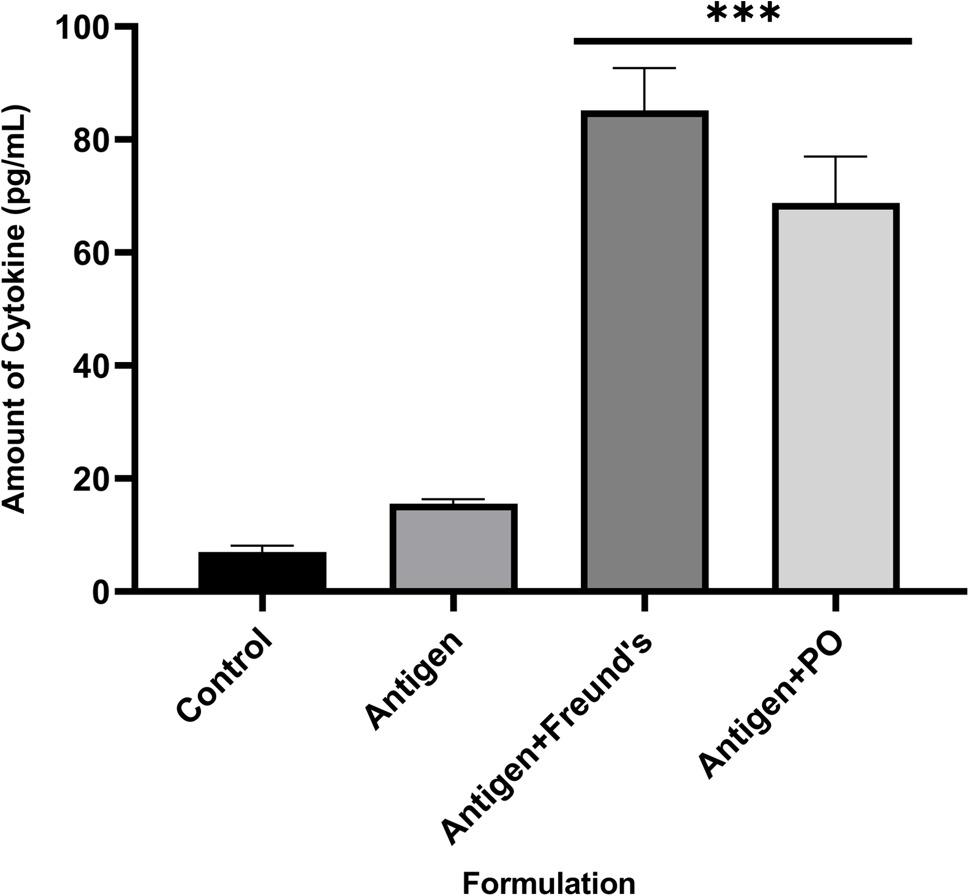
GM-CSF cytokine levels in J774 macrophage cells

The GM-CSF cytokine levels produced by J774 macrophage cells in response to the formulations are presented in Fig. 8. Data analysis showed that the combination of Antigen with Freund’s resulted in the highest GM-CSF production. Macrophages treated with the Antigen+Freund’s formulation exhibited a 12.17-fold increase in GM-CSF levels compared to the control group (*p* < 0.001).

### Evaluation of antibody titers after immunization

**Fig. 9:**
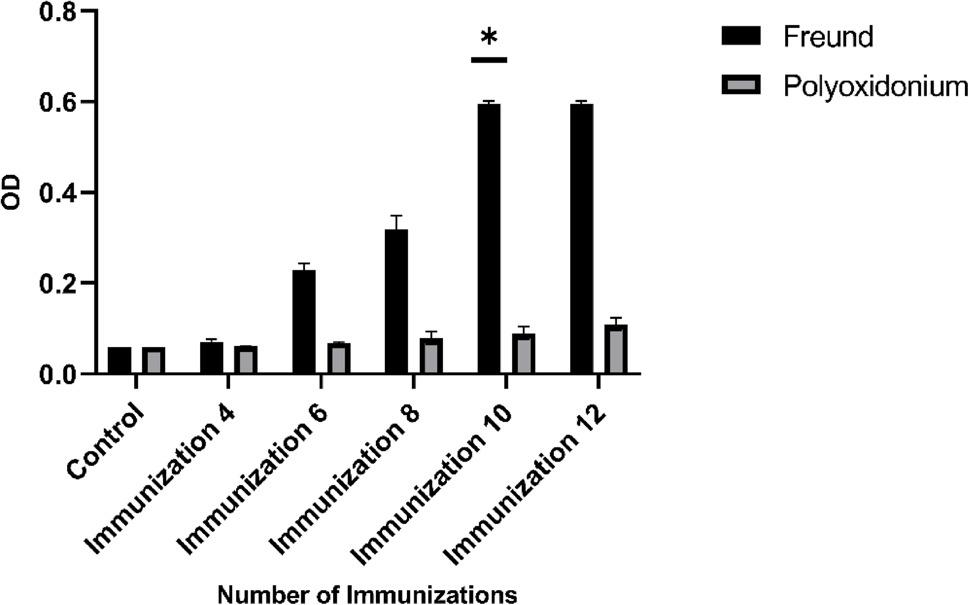
Determination of serum antibody titers in mice immunized with combinations including breast cancer antigens and Complete Freund’s adjuvant or PO adjuvant

To evaluate the in vivo immunostimulatory performance of the selected formulations, Balb/c mice were vaccinated with the antigen–adjuvant mixtures described above. Antibody responses against sonicated breast cancer antigens were markedly increased in immunized mice compared with the control group when the antigens were administered together with Complete Freund’s adjuvant or PO. The most pronounced increase was observed in the group immunized with sonicated antigens combined with Complete Freund’s adjuvant. As shown in Fig. 9, initial ELISA values measured in sera from the control group ranged between 0.05 and 0.06, whereas negative-control values ranged between 0.04 and 0.05. The negative-control values corresponded to pre-immune serum samples collected before the first immunization and were used to define baseline antigen-specific reactivity. After the fifth vaccination, antibody levels began to increase in the Complete Freund’s adjuvant group. Following the tenth vaccination with the antigen–Complete Freund’s adjuvant combination, specific antibody levels against breast cancer antigens were approximately 10-fold higher than those in the control group (*p* < 0.05). The marked increase detected in serum samples from this group was considered sufficient to proceed with hybridoma generation. In contrast, only a 2-fold increase in antibody levels was observed after the eleventh vaccination of antigens administered with PO adjuvant (Fig. 9). These findings indicate that Complete Freund’s adjuvant was more effective than PO in enhancing the immunogenicity of the breast cancer antigen preparation.

### Antibody levels in hybridoma culture supernatants

**Fig. 10:**
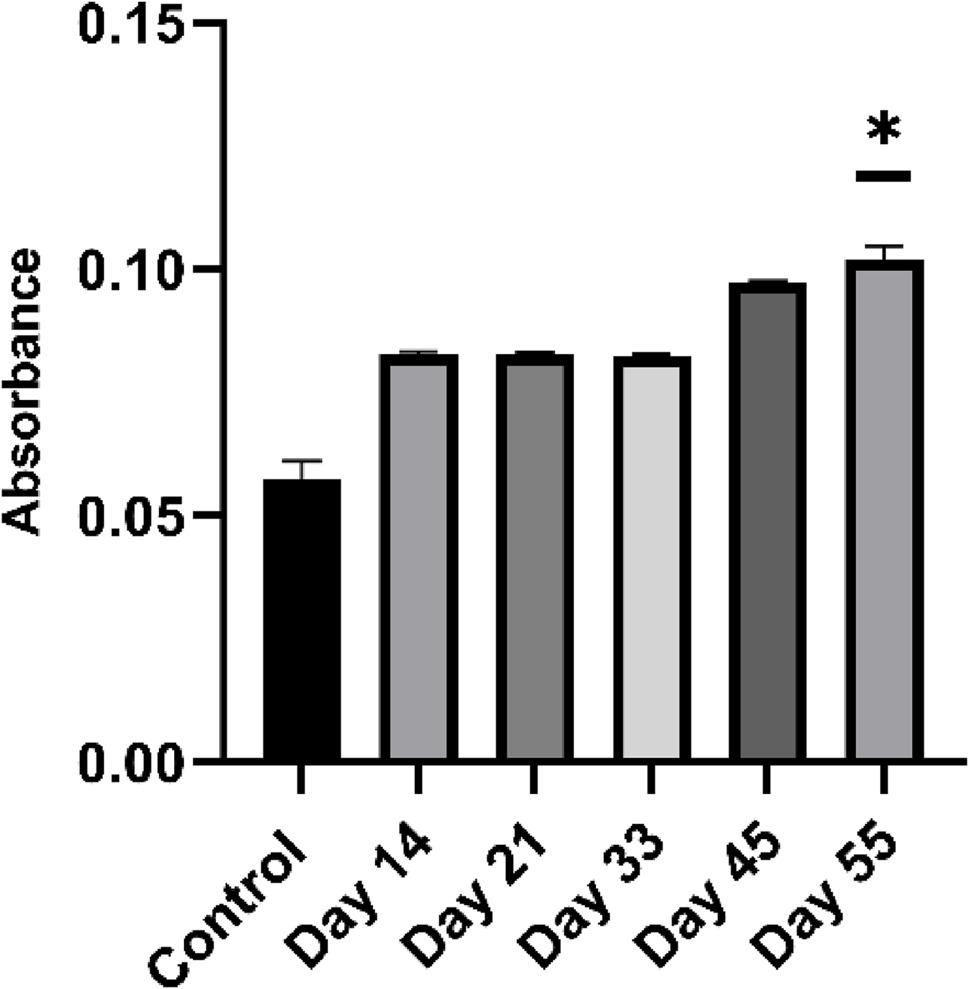
Antibody levels measured in supernatant samples of pooled hybridoma cultures

After the highest serum antibody response was obtained in mice immunized with the antigen and Complete Freund’s adjuvant formulation, spleens from the selected immunized mice were collected following euthanasia under ketamine–xylazine anesthesia and cervical dislocation. Splenocytes were then isolated and fused with myeloma cells to generate hybridoma cultures, which were followed for 55 days. Antibody production against breast cancer antigens was evaluated by ELISA using culture supernatants collected on days 14, 21, 33, 45, and 55 after fusion (Fig. 10). Antibody levels gradually increased during the culture period and reached their highest value on day 55. Compared with day 14, the antibody signal on day 55 was approximately 1.25-fold higher (*p* < 0.05). In addition, the day-55 antibody level was nearly two-fold higher than the control value. These findings indicate that antibody secretion by hybridoma cultures increased over time under the present culture conditions. Since single-cell cloning was not performed, these values represent the overall antibody output of pooled hybridoma populations.

### Comparison of hybridoma-derived antibody mixture and commercial antibodies in ELISA

**Fig. 11:**
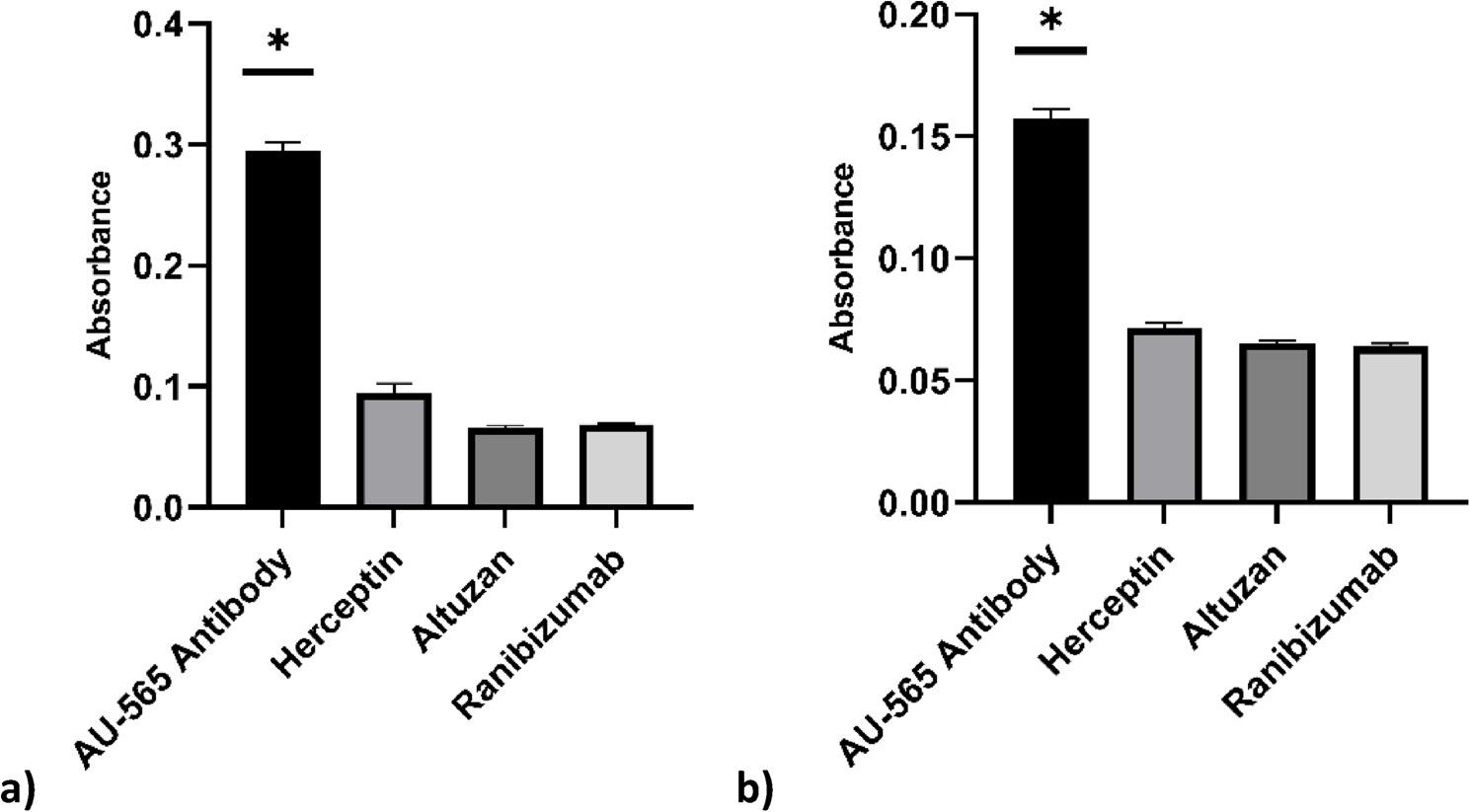
ELISA signals obtained using hybridoma-derived antibody mixture and commercial antibodies (**A**) AU-565 antigen (**B**) MCF-7 antigen

The ELISA performance of the hybridoma-derived antibody mixture and commercially available antibodies was compared using AU-565 and MCF-7 whole-cell lysates as coating antigens. Antibody-free wells containing coated antigen but no primary antibody were used as blank/negative controls to determine background absorbance. The hybridoma-derived antibody mixture exhibited a 2.9-fold higher ELISA signal against AU-565 antigens compared to the commercial antibody (*p* < 0.05). Similarly, the hybridoma-derived antibody mixture showed a 2.3-fold higher ELISA signal against MCF-7 antigens (*p* < 0.05) (Fig. 11). These results indicate that the pooled hybridoma antibody preparation exhibited stronger relative binding reactivity toward antigens derived from both AU-565 and MCF-7 breast cancer cell lines under the ELISA conditions used. The higher ELISA signal may reflect broader epitope recognition by the pooled hybridoma antibody mixture in the context of whole-cell lysates containing diverse tumor-associated antigens. However, because affinity, isotype composition, total immunoglobulin content, and reactivity against non-cancerous or unrelated control lysates were not evaluated in the current study, the stronger signal should be interpreted as preliminary evidence of relative lysate reactivity rather than definitive proof of superior specificity or diagnostic performance.

## Discussion

Many treatment approaches are currently being developed against breast cancer. However, it is believed that immunization of the body against breast cancer could be more powerful for prevention of this highly lethal disease [33]. For this purpose, vaccine formulations that can create immunity against many types of breast cancer have been tried to be developed, so far. Whole tumor cell lysates, immunogenic synthetic peptides derived from tumor antigens, DNA-based and dendritic cell-based vaccines are the most frequently used tools for stimulation of immunity against breast cancer [34]. Despite many advantages, there are some limitations of biotechnological cancer vaccine candidates [35]. For instance, most peptide vaccines are only influential over cancer types that are positive for human leukocyte antigen (HLA) and therefore patients who do not express common HLA classes cannot be treated with the vaccine [36]. Hence, there is a huge necessity for development of a comprehensive vaccine that could cover all breast cancer subtypes in general. Accordingly, using whole cell lysates including entire immunogenic cancer antigens is one of the most ideal vaccine approach to combat breast cancer. The AU-565 cell line was selected due to its well-characterized HER2-positive phenotype, which is known to be highly immunogenic and clinically relevant in breast cancer subtyping and targeted immunotherapy approaches. The AU-565 cell line represents a clinically relevant HER2-amplified breast cancer model and has been widely used in studies investigating HER2-driven oncogenic signaling and targeted therapy response. HER2-positive tumors remain a major breast cancer subtype with distinct biology and therapeutic considerations, and AU-565 has been reported to display a HER2-positive, hormone receptor-negative phenotype, supporting its suitability for antigen source selection in immunoassay-oriented antibody development [37, 38]. The broader translational relevance of this approach may extend to other HER2-positive breast cancer models beyond AU-565. If similar lysate-derived antigen complexity is preserved, hybridoma-derived antibody mixtures generated against HER2-amplified cell lines such as SK-BR-3 or BT-474 may also support broader immunoassay-oriented detection strategies, although this will require direct validation in future studies. Vaccination with whole cell cancer lysates could trigger the formation of specific antibodies against various antigens and substantial acquired immunity can be provided against breast cancer. In previous studies, experimental animals were exposed to peptides of breast cancer proteins in conjunction with Incomplete Freund adjuvant (IFA) and successful outcomes were achieved [39–41]. The choice of adjuvant is very substantial in the emergence of humoral and cellular immune response. Although many adjuvants could activate either Th1 or Th2 response, Complete Freund’s adjuvant (CFA) is an efficient instrument for stimulation of both Th1 and Th2 cells [42]. However, Complete Freund adjuvant has some disadvantages due to its toxic effect. In recent years, the use of more effective, non-toxic adjuvants have attracted attention in vaccine studies. For this purpose, many targeted vaccine delivery vehicles have been designed and polymeric adjuvants capable of regional degradation have also started to be used in vaccine applications. In one of the vaccine study against Leishmaniasis, it was reported that PO adjuvant was highly effective in regards to enhancement of immunogenic features of antigens used together while demonstrating no toxicity and therefore this adjuvant was suggested to be utilized in vaccine researches [27]. Polyoxidonium (azoximer bromide) has been utilized as an immunomodulatory compound and vaccine adjuvant in clinical and experimental settings, with reports highlighting its long-term use and favorable tolerability profile [43]. Polymer-based adjuvants such as azoximer bromide have been investigated for their ability to support immune activation while potentially reducing toxicity-associated constraints observed with classical strong adjuvants. Therefore, comparing CFA with PO may provide useful insight into balancing potency and safety in whole-tumor antigen immunization strategies [31]. Accordingly, this study compared the effectiveness of Complete Freund’s adjuvant and Polyoxidonium (PO) in generating strong humoral immune responses against AU-565 whole-cell lysates and in supporting the production of a hybridoma-derived antibody mixture for breast cancer-oriented immunoassay applications. We initially evaluated the secreted nitric oxide amounts from macrophages treated with antigen and adjuvant combinations. This assay was followed with the calculation of in vivo antibody levels in mice immunized with different vaccine formulations prepared with two distinct adjuvants and their immunostimulatory performances were compared. The vaccine formulation with higher scores was selected and examined for further studies including evaluation of the ELISA reactivity of the hybridoma-derived antibody mixture. According to the results obtained, whole-cell lysates prepared by sonication successfully induced antibody production in immunized mice regardless of the adjuvant type. However, the formulation containing Complete Freund’s Adjuvant (CFA) led to substantially higher antibody titers than the Polyoxidonium (PO) formulation, supporting the selection of the CFA-based immunogen for subsequent hybridoma experiments. Following cell fusion and selection, antibody levels measured in pooled hybridoma culture supernatants increased over the incubation period, indicating sustained antibody accumulation during long-term culture. A potential limitation of this study is the small sample size used in the in vivo immunization experiments (*n* = 3 per group), which may limit statistical power; nevertheless, consistent antibody titer trends and cytokine response patterns across replicates support the reliability of the observed immune activation. In addition, cytokine profiling demonstrated that the CFA-based formulation strongly upregulated IL-12, TNF-α, and GM-CSF in macrophage cultures, indicating a pronounced pro-inflammatory response pattern, while a moderate IL-6 response suggested concurrent support for humoral activation. Since these data were generated in an in vitro macrophage model, they should be interpreted as preliminary mechanistic evidence rather than definitive proof of systemic adaptive immune polarization in vivo. Together, these findings indicate that the selected formulation enhanced antibody generation and induced a measurable pro-inflammatory response pattern in the macrophage model, which may support its relevance for breast cancer-oriented immunoassay development.

The hybridoma-derived antibody mixture obtained in this study may provide utility in breast cancer-related immunoassay applications due to its strong reactivity toward tumor lysate antigens. In breast cancer diagnosis, commonly used methods such as mammography, ultrasound, and biopsy remain indispensable; however, they may require time-consuming workflows and, in the case of biopsy, invasive intervention. In contrast, antibody-mediated serological assays enable more rapid screening by capturing tumor-associated antigens directly in clinical samples. An important advantage of using an antibody preparation with multi-epitope recognition is the potential for broader antigen coverage and higher functional avidity when targeting heterogeneous tumor antigens [8]. In our ELISA comparisons, the hybridoma-derived antibody mixture generated against AU-565 lysates produced stronger signals than the commercial antibody and also reacted with MCF-7 lysates, which may be consistent with recognition of shared breast cancer-associated epitopes across subtypes. Because the antibody preparation was derived from pooled hybridoma populations without single-cell cloning, the relative contribution of individual antibody-secreting clones remains undefined. Therefore, culture-dependent shifts in clone composition cannot be excluded and may affect long-term stability and inter-batch reproducibility of the antibody mixture. Accordingly, the present antibody preparation should be interpreted as a proof-of-concept oligoclonal hybridoma product rather than a finalized analytical reagent. Since no direct comparison was performed with isolated monoclonal hybridoma clones, the current study cannot determine whether the observed broader reactivity reflects a true functional advantage of oligoclonality or a variable composite profile arising from pooled culture conditions. Nevertheless, ELISA signal intensity alone does not establish superior analytical specificity, affinity, or functional performance. Since isotype profiling, normalization to total antibody concentration, and testing against non-cancerous or unrelated control lysates were not performed, these findings should be interpreted as preliminary evidence of broader lysate reactivity rather than definitive diagnostic superiority. Although these findings support preliminary immunoassay relevance, further characterization—such as binding affinity (e.g., Kd), analytical sensitivity/specificity, and cross-reactivity against non-cancerous controls—should be performed before any clinical or diagnostic reliability can be inferred. Future studies should also validate performance using orthogonal techniques such as immunohistochemistry, flow cytometry, and surface plasmon resonance, and should incorporate in vivo cytokine profiling to better reflect systemic immune modulation following vaccination.

From a translational perspective, whole-tumor antigen platforms (including whole-cell lysates) continue to attract interest in therapeutic cancer vaccine research due to their ability to provide broad antigen repertoires, potentially mitigating escape mechanisms arising from antigen loss. Recent literature emphasizes that whole-tumor antigen vaccination approaches remain a promising area, although optimization of immune activation, formulation design, and assay validation are essential for clinical impact [41]. Within this framework, our findings support the utility of AU-565 lysate-based immunogens and pooled hybridoma antibody mixtures as practical tools for breast cancer antigen capture and immunoassay development. For diagnostic translation, the next step will be analytical validation under standardized immunoassay conditions, including determination of intra-/inter-assay variability, limit of detection (LOD), and receiver operating characteristic (ROC) performance using clinically relevant sample sets. Such assay-level validation will be essential to define sensitivity, specificity, and cut-off thresholds required for clinical decision-making.

Limitations of the study include the small in vivo sample size (*n* = 3 per group), which limits statistical power and reduces the strength of biological generalization, as well as the use of pooled hybridoma supernatants without single-cell cloning and the lack of affinity/avidity measurements (e.g., Kd) using biophysical methods. Because pooled hybridoma cultures may change in composition over time, clonal stability and batch-to-batch reproducibility could not be resolved in the current study. In addition, no direct comparison was performed with isolated monoclonal hybridoma clones, limiting conclusions regarding whether oligoclonality confers a true functional advantage beyond pooled-culture variability. Another limitation is that immune polarization was inferred primarily from macrophage-derived NO and cytokine readouts. Ex vivo splenocyte restimulation, T-cell-oriented cytokine assessment, and serum cytokine confirmation were not performed; therefore, the adaptive in vivo immune response could not be directly resolved in the current study. In addition, assay-level diagnostic validation parameters such as sensitivity/specificity, limit of detection, and performance in clinical matrices were not assessed in the current study. These aspects should be addressed in follow-up studies to strengthen clinical translation. Stronger ELISA signals obtained with the pooled hybridoma-derived antibody mixture cannot be interpreted as direct evidence of superior specificity or functional superiority, because affinity estimation, isotype profiling, and testing against unrelated or non-malignant lysates were not included in the current study. Moreover, the total protein yield of individual AU-565 lysate preparations and endotoxin contamination were not independently assessed, which should be addressed in future studies to further standardize antigen preparation. Accordingly, the in vivo findings should be interpreted as preliminary and will require confirmation in larger animal cohorts in future studies.

## Conclusion

Consequently, sonicated breast cancer lysates obtained from the AU-565 cell line were combined with two different adjuvants to develop experimental vaccine formulations. The in vitro immunostimulatory efficacy of these formulations was supported by their ability to significantly increase nitric oxide (NO) production in macrophage cells, while in vivo immunogenicity was confirmed by elevated serum antibody titers following immunization. Among the tested formulations, the combination of AU-565 antigens with Complete Freund’s Adjuvant (CFA) induced stronger humoral responses and higher antibody titers compared with the Polyoxidonium (PO)-based formulation.

Following immunization, hybridoma cultures were generated from splenocytes of mice vaccinated with the CFA-supported antigen formulation. Since single-cell cloning was not performed, antibodies collected from culture supernatants represent a hybridoma-derived antibody mixture produced by pooled hybridoma populations. This pooled antibody preparation accumulated over long-term incubation and demonstrated robust binding reactivity. Importantly, the hybridoma-derived antibody mixture reacted strongly not only with AU-565 lysates but also with MCF-7 breast cancer lysates, suggesting recognition of shared tumor-associated epitopes across different breast cancer subtypes. Nevertheless, because the antibody mixture originated from pooled hybridoma populations without clonal isolation, its long-term compositional stability and batch reproducibility remain unresolved. In the absence of direct comparison with isolated monoclonal clones, the observed broader reactivity should therefore be interpreted as proof-of-concept rather than definitive evidence of a functional advantage of oligoclonality.

Although these findings support preliminary diagnostic and translational relevance, further biochemical and analytical characterization—including binding affinity (e.g., Kd), isotype composition, normalization to antibody content, specificity, sensitivity, and cross-reactivity against non-cancerous controls—should be performed in future studies before clinical or diagnostic reliability can be established. To the best of our knowledge, this is the first study demonstrating the immunostimulatory capacity of AU-565-derived antigens prepared via sonication, and our findings indicate that this capacity can be further enhanced by incorporating suitable adjuvants. While CFA exhibited superior performance in eliciting antibody responses, PO also promoted macrophage-derived NO release, supporting its potential as a safer immunomodulatory alternative.

Finally, cytokine analysis strengthened the immunological interpretation of the vaccine formulations. The CFA-supported antigen formulation induced significantly increased levels of IL-12, GM-CSF, and TNF-α, consistent with a pro-inflammatory response pattern in the macrophage model, while a moderate IL-6 response suggested concurrent support for humoral pathways. However, confirmation of adaptive immune polarization will require dedicated ex vivo and in vivo immune profiling. Overall, these results indicate that AU-565-derived whole-cell lysates, particularly when combined with CFA, represent a promising platform for future breast cancer-oriented immunodiagnostic and experimental immunotherapy research.

Future studies should include single-clone isolation of dominant hybridoma populations to directly compare monoclonal and pooled antibody preparations in terms of signal intensity, specificity, clonal stability, and inter-batch reproducibility, together with affinity/avidity measurements using techniques such as SPR or biolayer interferometry. In addition, orthogonal validation using immunohistochemistry and flow cytometry on breast cancer models, including non-cancerous controls, will be essential to determine analytical specificity and translational diagnostic accuracy. Additionally, validation using clinically relevant sample sets and ROC-based cut-off determination will be essential to define diagnostic performance for real-world applications.

## Data Availability

The datasets generated and/or analysed during the current study are available from the corresponding author on reasonable request.

## Abbreviations

AR: Androgen receptor
ATCC: American Type Culture Collection
BLI: Biolayer interferometry
CFA: Complete Freund’s Adjuvant
CK5/6: Cytokeratin 5/6
DMSO: Dimethyl sulfoxide
EGFR: Epidermal growth factor receptor
ELISA: Enzyme-linked immunosorbent assay
ER: Estrogen receptor
FBS: Fetal bovine serum
GM-CSF: Granulocyte–macrophage colony-stimulating factor
HAT: Hypoxanthine–aminopterin–thymidine
HER2: Human epidermal growth factor receptor 2
HLA: Human leukocyte antigen
HT: Hypoxanthine–thymidine
IFA: Incomplete Freund’s adjuvant
IL-6: Interleukin-6
IL-12: Interleukin-12
Kd: Equilibrium dissociation constant
Ki-67: Proliferation marker Ki-67
LOD: Limit of detection
mAb: Monoclonal antibody
MTT: 3-(4,5-dimethylthiazol-2-yl)-2,5-diphenyltetrazolium bromide
NO: Nitric oxide
PBS: Phosphate-buffered saline
PEG: Polyethylene glycol
Pen-Strep: Penicillin–streptomycin
PO: Polyoxidonium
PR: Progesterone receptor
ROC: Receiver operating characteristic
RPMI-1640: Roswell Park Memorial Institute 1640 medium
SPR: Surface plasmon resonance
TNF-α: Tumor necrosis factor-alpha
UV-Vis: Spectrophotometry
